# Fluctuating Hemiparesis Secondary to Moyamoya Phenomenon in a Child with Down Syndrome: a case report

**DOI:** 10.1186/1757-1626-1-240

**Published:** 2008-10-15

**Authors:** Richard A Rison

**Affiliations:** 1 Clinical Assistant Professor of Neurology, University of Southern California, Keck School of Medicine, Los Angeles County Medical Center, Neurology Consultants Medical Group, 12291 E. Washington Blvd. Suite #303, Whittier, California, 90606, USA

## Abstract

Moyamoya phenomenon is a term used to describe extensive collateralization of the circle of Willis arteries associated with severe unilateral or bilateral internal carotid artery stenosis or occlusion in the presence of certain conditions. Down syndrome is among these conditions. A case is reported of a young girl with Down syndrome who presented with fluctuating right-sided weakness and facial droop found to have cerebral ischemia. Subsequent investigations disclosed characteristic "puff of smoke" patterns on angiographic studies consistent with moyamoya phenomenon. The patient was initially treated with aspirin and eventually underwent an encephalomyosynangiosis. This young patient with Down syndrome and moyamoya phenomenon serves as a reminder of the association between these two conditions.

## Background

Moyamoya disease is a nonatherosclerotic and noninflammatory condition characterized by progressive stenosis of the terminal internal carotid artery and the proximal portions of the anterior cerebral and middle cerebral arteries. The term "moyamoya disease" is used when the internal carotid artery stenosis and associated collaterals are observed bilaterally and when no associated diseases are identified. "Moyamoya phenomenon" is used to describe the extensive collateralization of the circle of Willis arteries associated with severe unilateral or bilateral internal carotid artery stenosis or occlusion in the presence of certain conditions. Clinical presentation is variable and age dependent, with risk of hemorrhage increasing with age and ischemic symptoms predominate in the young [1]. There have been published case reports of the co-occurrence of both moyamoya and Down syndrome, but knowledge regarding the etiology is sparse.

## Case presentation

A 4 and 1/2 year old Mexican girl with Down syndrome presented to the emergency department of a local tertiary care center with symptoms of fluctuating right arm weakness and difficulty smiling that started approximately 4 hours before admission. Her parents found her in bed that morning and noted that she had difficulty playing with one of her dolls because she kept dropping them with her right hand. Her smile also seemed a bit "twisted" according to her mother, and she walked with a limp on her right leg. There was no reported headache or vomiting nor did the child have any history of seizures.

On examination she was normotensive and afebrile. The child was sitting comfortably on the stretcher with her mother present beside her. The child had typical facial characteristics of trisomy 21. Her speech was delayed. Cranial nerve testing revealed a mild right-sided lower facial droop. Motor testing revealed a fluctuating 4+/5 MRC (Medical Research Council) Grade strength of her right upper extremity and right lower extremity. Long-tract signs included an equivocal right toe and a down-going left toe. She walked towards her mother with a slight limp favoring the right side.

**Figure 1:**
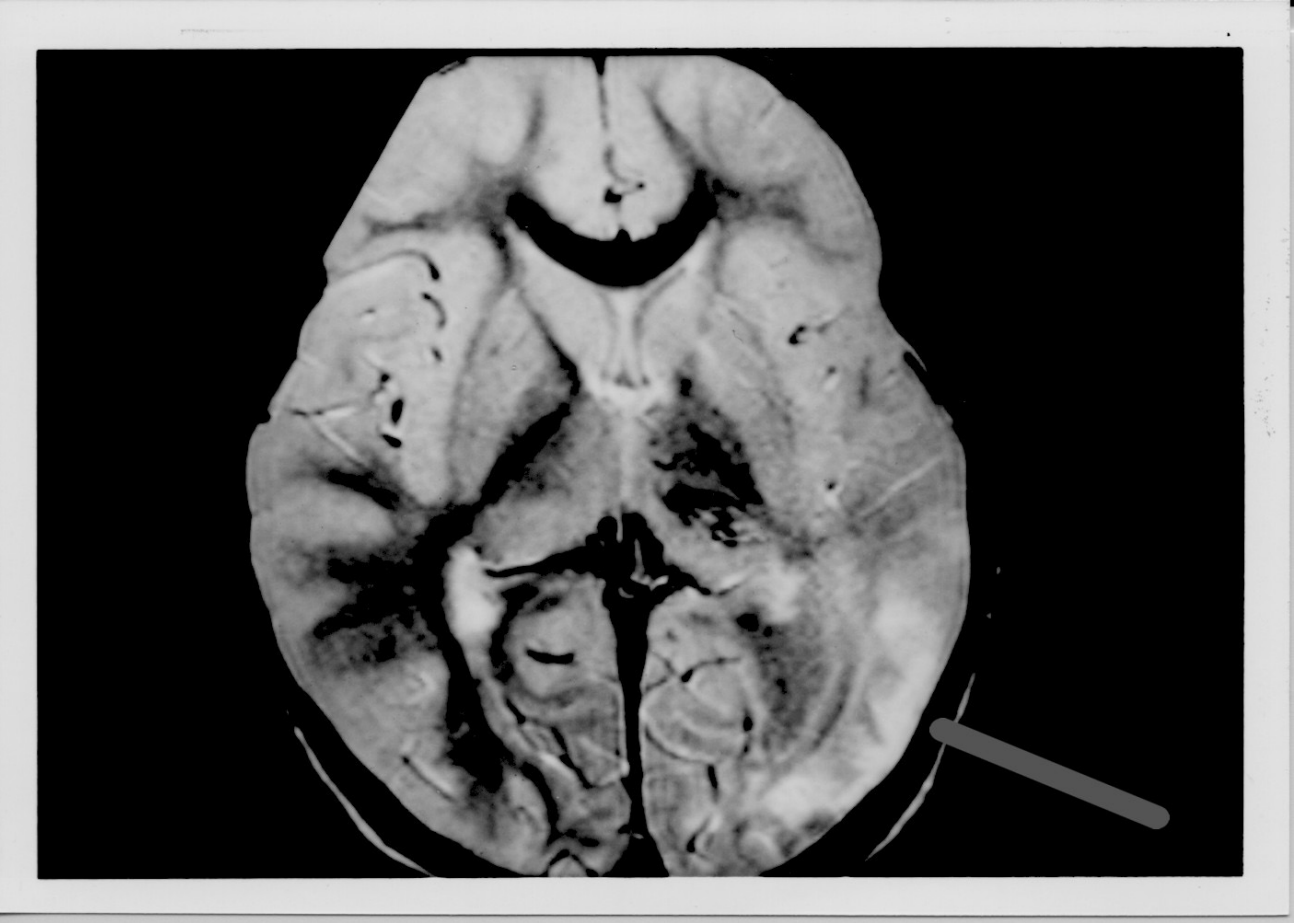
Magnetic resonance imaging obtained the day after admission demonstrating acute ischemia in the left temporal and parietal area (horizontal gray bar).

**Figure 2:**
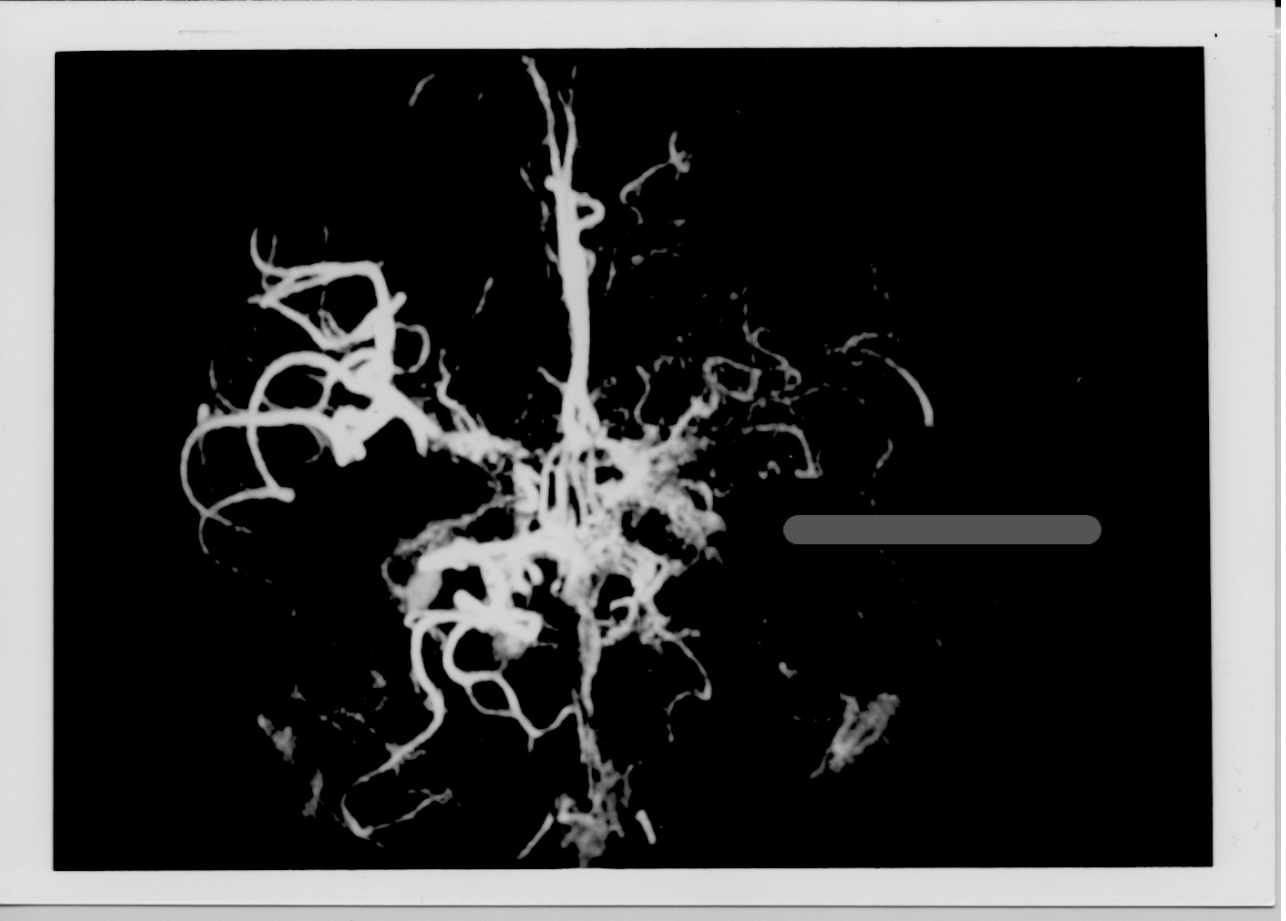
Magnetic resonance angiogram study obtained the day after admission demonstrating prominent collaterals and occlusions in the region of the middle cerebral arteries also involving the supraclinoid region (horizontal gray bar).

Routine laboratory serum studies were unremarkable. Initial head computed tomography did not reveal any acute findings. Subsequent magnetic resonance angiography and imaging revealed acute ischemic changes in the left temporal-parietal area with left middle cerebral artery collateralization (see Figures 1 and 2, respectively).

The differential diagnosis included transient ischemic attacks, ischemic stroke, dissection, hypoglycemia, cardiac source emboli, hypercoaguable states, seizures, and migraines [1].

The patient was given aspirin 81 mg po qd and physical therapy. She was discharged home and eventually underwent an an encephalomyosynangiosis.

Her initial outcome post-operatively was satisfactory but unfortunately she was lost to follow-up.

## Discussion

Moyamoya disease is a noninflammatory, non-vasculitic and nonatherosclerotic condition characterized by progressive stenosis of intracranial arteries. The intracranial arteries usually involved are the terminal internal carotid artery and the proximal portions of the anterior cerebral and middle cerebral arteries. There is a slowly progressive occlusion which permits the development of the unique small anastomotic collateral pathways causing the "puff of smoke" appearance on the angiogram or "cerebral basal rete mirabile". Moyamoya disease is the term used when the internal carotid artery stenosis and associated collaterals are observed bilaterally and when no associated diseases are identified (i.e., idiopathic). Moyamoya phenomenon is the term used to describe extensive collateralization of the circle of Willis arteries associated with severe unilateral or bilateral internal carotid artery stenosis or occlusion in the presence of certain conditions [1].

The list of conditions and causes associated with moyamoya phenomenon is long an includes neurofibromatosis, craniopharyngioma, optic glioma, distal internal carotid artery compression by tumor, chronic meningitis, leptospiral infections, atherosclerosis, sickle cell disease, antiphospholipid syndrome or lupus anticoagulant, Alagille syndrome (an autosomal dominant disorder associated with abnormalities of the liver, heart, skeleton, eye, and kidneys and characteristic facial morphology, osteogenesis imperfecta, Costello syndrome (a rare genetic disorder that affects multiple organs), and cocaine use. Moyamoya phenomenon has also been associated with extracranial and peripheral arterial disease, essential thrombocythemia, and birth control pills (possibly in combination with cigarettes). Cases of moyamoya vessels developing around the stenosis of a vessel supplying an arteriovenous malformation have been reported. Recent case reports suggest an association between thyrotoxicosis (Grave disease) and severe moyamoya disease [Please see reference 1 for an excellent review].

Moyamoya was first described in the late 1950's with most cases being reported from Japan. Clinical presentation is variable and age dependent. In childhood strokes tend to be ischemic, whereas in adults they are more often hemorrhagic. It occurs with a bimodal onset, peaking in the first and fourth decades of life. Children are more likely to present with acute transitory hemiplegia secondary to recurrent cerebrovascular episodes [2]. Down syndrome in association with moyamoya has been reported since the 1980's and ischemic vascular pathologies in general are common. Infact, new-onset focal weakness is not uncommon in Down syndrome and moyamoya syndrome is one of the more common causes [3].

The exact etiology of moyamoya is unknown. It has been speculated that there is a genetic mode of inheritance based on a higher incidence of the disease in Asian populations and on familial occurrence in both Asians and whites and this has been supported by genetic linkage analysis. Various connective tissue, angiogenesis, and inflammatory mediators have been implicated including fibroblast growth factor, transforming growth factor β_1_, elastin, and prostaglandin E_2 _[4].

The association of moyamoya with Down syndrome is also not fully understood. The incidence of moyamoya syndrome in patients with trisomy 21 is approximately three times the general population [5]. Moyamoya is characterized by elastopathic vascular damage, and patients with Down syndrome are in general predisposed to vascular diseases, including abnormal nail bed capillary morphology, high pulmonary vascular resistance, retinal vessel abnormalities, renovascular hypertension, and primary intimal fibroplasias [5,6]. There has also been a reported association of elastosis perforans serpiginosa (a dermatologic disease characterized by transepithelial elimination of abnormal elastic fibers, and focal dermal elastosis) with both Down syndrome and moyamoya [7]. Chromosome 21 encodes proteins that affect arterial physiology and elasticity, including superoxide dismutase 1, interferon γ receptor, cystathionine β-synthetase, and collagen type IV (found in the intima of large arteries). It has been postulated that these proteins may be abnormally expressed when three copies of chromosome 21 are present [2]. Pathologic studies are limited but support this notion [8].

Various treatments have been explored, although none are ideal. Medical therapy in ischemic moyamoya conditions include aspirin and hydration [2]. Surgical treatment includes both direct and indirect revascularization procedures, and one review concludes no difference between the two techniques [9]. The largest and most recent study to date of long-term outcome of surgical revascularization in Downs syndrome patients with moyamoya (ages ranging from 1 to 29 years old) suggests that clinical, radiologic, and angiographic features of moyamoya phenomenon associated with Down syndrome are comparable to those of idiopathic moyamoya disease. Cerebral revascularization surgery with the pial synangiosis technique seems to confer long-lasting protection against additional strokes in this patient population [10].

In summary, the presence of moyamoya syndrome should be considered in the evaluation of patients with Down syndrome who present with transient ischemic attack-like symptoms of fluctuating hemiparesis even in the very young. Surgical revascularization with encephalomyosynangiosis apparently confers long-term benefits.

## Conclusion

The presence of moyamoya syndrome should be considered in the evaluation of patients with Down syndrome who present with transient ischemic attack-like symptoms of fluctuating hemiparesis even in the very young. Surgical revascularization with encephalomyosynangiosis may confer long-term benefits.

## Consent

Written informed consent was obtained from the patients' mother for publication of this case report and accompanying images. Please note that an institution-specific consent form was used and dated from Children's Hospital in Los Angeles, California, USA. A copy of the written consent is available for review by the Editor-in-Chief of this journal.

## Competing interests

The author declares that they have no competing interests.

## Authors' contributions

RAR wrote the entire case report.

## References

[B1] Singhal AB, Gilman S Moyamoya disease. MedLink Neurology.

[B2] Boggs S, Hariharan SL (2008). An uncommon presentation of stroke in a child with trisomy 21. Pediatr Emerg Care.

[B3] Worley G, Shbarou R, Heffner AN, Belsito KM, Capone GT, Kishnani PS (2004). New onset focal weakness in children with Down syndrome. Am J Med Genet A.

[B4] Gosalakkal JA (2002). Moyamoya disease: a review. Neurol India.

[B5] De Borchgrave V, Saussu F, Depre A, de Barsy T (2002). Moyamoya disease and Down syndrome: a case report and review of the literature. Acta Neurol Belg.

[B6] Fung CW, Kwong KL, Tsui EY, Wong SN (2003). Moyamoya in a child with Down syndrome. Hong Kong Med J.

[B7] Espinosa PS, Baumann RJ, Vaishnav AG (2008). Elastosis perforans serpiginosa, Down syndrome, and moyamoya disease. Pediatr Neurol.

[B8] Watabe N, Nishino A, Arai H, Nishimura S, Suzuki S, Uenohara H, Sakurai Y, Suzuki H (2005). An autopsy case of Down's syndrome with moyamoya syndrome. No Shinkei Geka.

[B9] Fung LW, Thompson D, Ganesan V (2005). Revascularisation surgery for paediatric moyamoya: a review of the literature. Childs Nerv Syst.

[B10] Jea A, Smith ER, Robertson R, Scott RM (2005). Moyamoya syndrome associated with Down syndrome: outcome after surgical revascularization. Pediatrics.

